# Understanding “Alert Fatigue” in Primary Care: Qualitative Systematic Review of General Practitioners Attitudes and Experiences of Clinical Alerts, Prompts, and Reminders

**DOI:** 10.2196/62763

**Published:** 2025-02-07

**Authors:** Illin Gani, Ian Litchfield, David Shukla, Gayathri Delanerolle, Neil Cockburn, Anna Pathmanathan

**Affiliations:** 1 Department of Health Sciences University of Birmingham Birmingham United Kingdom; 2 Population Health Sciences Centre for Academic Primary Care University of Bristol Bristol United Kingdom

**Keywords:** primary care, general practitioners, alert fatigue, computer decision support systems, fatigue, qualitative, systematic review, quality of care, clinical behaviors, behaviors, database, family practice, family, algorithm, patient safety, patient

## Abstract

**Background:**

The consistency and quality of care in modern primary care are supported by various clinical reminders (CRs), which include “alerts” describing the consequences of certain decisions and “prompts” that remind users to perform tasks promoting desirable clinical behaviors. However, not all CRs are acted upon, and many are disregarded by general practitioners (GPs), a chronic issue commonly referred to as “alert fatigue.” This phenomenon has significant implications for the safety and quality of care, GP burnout, and broader medicolegal consequences. Research on mitigating alert fatigue and optimizing the use of CRs remains limited. This review offers much-needed insight into GP attitudes toward the deployment, design, and overall effectiveness of CRs.

**Objective:**

This systematic review aims to synthesize current qualitative research on GPs’ attitudes toward CRs, enabling an exploration of the interacting influences on the occurrence of alert fatigue in GPs, including the deployment, design, and perceived efficacy of CRs.

**Methods:**

A systematic literature search was conducted across the Health Technology Assessment database, MEDLINE, MEDLINE In-Process, Embase, CINAHL, Conference Proceedings Citation Index, PsycINFO, and OpenGrey. The search focused on primary qualitative and mixed methods research conducted in general or family practice, specifically exploring GPs’ experiences with CRs. All databases were searched from inception to December 31, 2023. To ensure structured and practicable findings, we used a directed content analysis of the data, guided by the 7 domains of the Non-adoption, Abandonment, Scale-up, Spread, and Sustainability (NASSS) framework, including domains related to Technology, Adopter attitudes, and Organization.

**Results:**

A total of 9 studies were included, and the findings were organized within the 7 domains. Regarding Condition and Value Proposition, GPs viewed CRs as an effective way to maintain or improve the safety and quality of care they provide. When considering the attributes of the Technology, the efficacy of CRs was linked to their frequency, presentation, and the accuracy of their content. Within Adopters, concerns were raised about the accuracy of CRs and the risk that their use could diminish the value of GP experience and contextual understanding. From an Organization perspective, the need for training on the use and benefits of CRs was highlighted. Finally, in the context of the Wider system and their Embedding Over Time, suggestions included sharing best practices for CR use and involving GPs in their design.

**Conclusions:**

While GPs acknowledged that CRs, when used optimally, can enhance patient safety and quality of care, several concerns emerged regarding their design, content accuracy, and lack of contextual nuance. Suggestions to improve CR adherence included providing coherent training, enhancing their design, and incorporating more personalized content.

**Trial Registration:**

PROSPERO CRD42016029418; https://www.crd.york.ac.uk/prospero/display_record.php?RecordID=29418

**International Registered Report Identifier (IRRID):**

RR2-10.1186/s13643-017-0627-z

## Introduction

**Figure 1:**
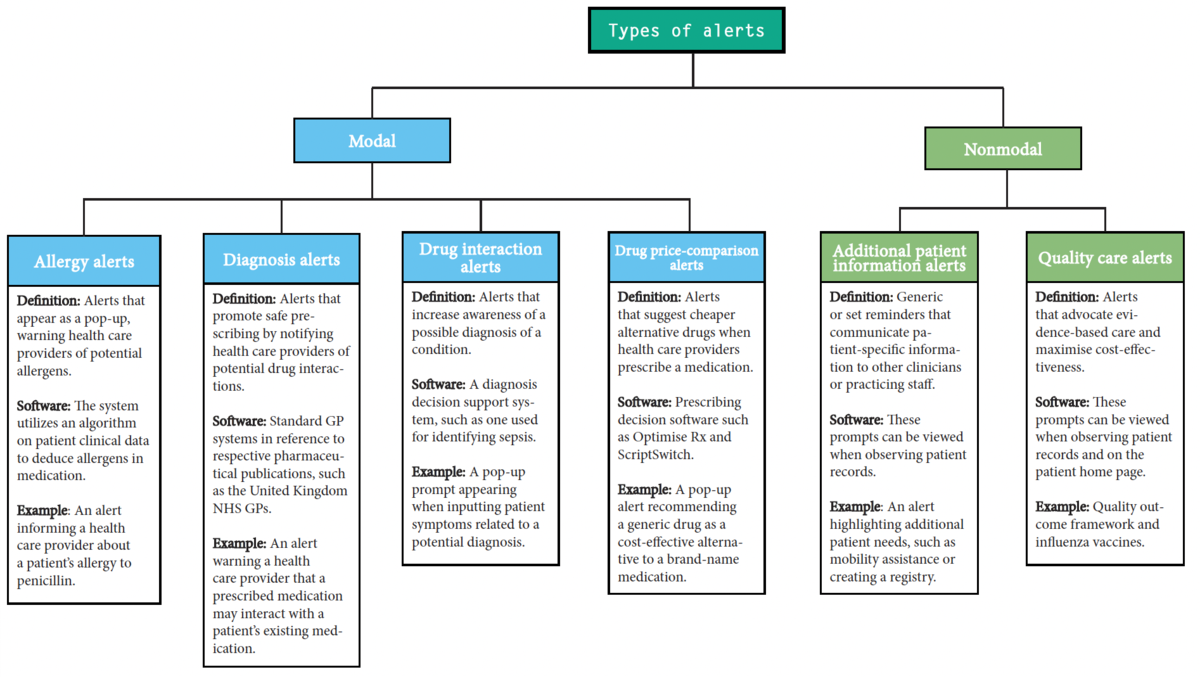
Types of clinical reminders and alerts used in clinical management systems in primary care (Cecil et al [11]).

Primary care is facing unprecedented pressure across the globe, caught between growing patient demand, issues of workforce recruitment and retention, and increasingly complex options for treatment [1,2]. To improve the consistency and quality of care in this dynamic and highly pressured environment, health systems worldwide have introduced guidelines for care informed by the latest clinical evidence [3,4]. These guidelines are promoted and supported in high-income countries through a range of clinical reminders (CRs) that are presented to general practitioners (GPs) via their clinical management systems and consist of “alerts” that remind them of the consequences of making a certain decision, and “prompts” that remind the user to perform a task that promotes desirable clinical behaviors [5-10]. These alerts can relate to potential diagnoses, drug-drug interactions, and allergies, and prompts linked to patient-specific needs, further information, and recommendations for high-quality care (see Figure 1; see also [11]).

The growing sophistication of medical knowledge and the electronic systems that manage patient data and inform clinical decision-making have led to a steady increase in the activation of CRs at the point of care and the cognitive load placed on GPs [12-15]. Not all CRs are acted upon, and some may justifiably be considered irrelevant or inapplicable [16,17], but increasingly GPs are disregarding pertinent or urgent CRs [18-20], a chronic negligence commonly referred to as “alert fatigue” [21].

Despite the implications of alert fatigue for the safety and quality of care, GP burnout, and the medico-legal consequences for individuals and their organizations, the proliferation of CRs in primary care is reliant on evidence limited to their effects on a specific condition [22,23]. What remains underexplored is how their use might be optimized in the context of the cumulative effect of multiple CRs on a single health care provider [19,20,22,24]. There is then a need to better understand GP attitudes toward the deployment, design, and overall effectiveness of CRs, as well as broader implications for their impact on patient-clinician communication [22,25]. The systematic review presented here aims to address this by synthesizing current qualitative research on GPs’ attitudes toward CRs describing the findings within the Non-adoption, Abandonment, Scale-up, Spread, and Sustainability (NASSS) framework [26]. This framework was designed to study unfolding digital health technology programs and so provides the opportunity for offering structured insight into the future design, use, and positioning of CRs in primary care.

## Methods

### Applying the NASSS Framework to Digital Health Implementation

The review used best practices in systematic review methodology and directed content analysis [27,28]. We describe the results through the lens of the NASSS framework developed to support, guide, and monitor the implementation of digital technologies in health and social care [26,29,30]. The framework uses 7 domains to describe various aspects of the technology, end users, organizational structure, and broader policy-driven context [26]. The domains are defined alongside their potential impact on implementation in Table 1.

**Table 1:** The NASSS^a^ framework: domains, definitions, and influences on implementation (adapted from [26,29-31]).

Domain	Definition	Influences on implementation
Condition	The condition(s) for which the innovation or technology has been designed. These can be physical, mental, or psychosocial in nature.	The complexity of the condition. Its metabolic volatility, association with comorbidities, and impact on cognitive function [32].
Technology	The technology/ies or other innovation that is/are being introduced. Include(s) both hardware and software and can include a novel protocol or pathway—or some combination of these.	Its material properties, functionality, dependability, and speed [33]. The knowledge needed to use them [34]. The knowledge generated by the technology [35]. The supply model and the relationship with the care provider [36]. The ownership of intellectual property [37].
Value Proposition	The value (financial or otherwise) that the new technology and care model generates. This includes, for commercial stakeholders, the return on investment; for patients, improvements in comfort or quality of life; for health care organizations, improvements in quality, safety, inclusivity, and efficiency of the care delivered.	Provision of value to a range of stakeholders, suppliers, patients, the health care system, and taxpayers or insurers. Formulating a credible business plan where efficacy or cost-effectiveness studies are unavailable or contested [38,39].
Adopters	The intended users of the technology or other innovation. This includes patients/laypersons, professionals, administrative, and support staff.	Acceptability to service users and their families/carers [40]. Attitudes toward new and emerging technologies [41]. Influence of sociocultural factors such as poverty or social exclusion [42]. Acceptability to staff. Impact on roles, professional traditions, and codes of conduct [26].
Organizations	The cultural and organizational characteristics of the organizations involved. This includes structure, capacity, and capability to adopt new ways of working, as well as resources of staff and infrastructure.	The organization’s general capacity to innovate [43]. Readiness for this particular technology [26]. The decisions around funding the intervention (including the presence of interorganizational agreements or speculative cross-system savings in the funding decision [44]). The extent of the change needed, including the potential disruption to existing routines [45]. The work required in implementation, including staff engagement, fidelity of implementation, and evaluation [46].
Wider System	The national and local context for the introduction of the technology or program	The impact of national and local policies and objectives [47]. The support of regulatory or professional bodies [48]. Sociocultural factors including public perceptions of the technology [49]. The presence of interorganizational networking and collaborative initiatives in supporting implementation [50].
Embedding Over Time	The key changes and uncertainties expected to affect the integration of the technology over the next 3-5 years.	The ability of the technology to adapt to changing contexts [51]. The resilience and cultural stability of the organizations involved [52].

^a^NASSS: Non-adoption, Abandonment, Scale-up, Spread, and Sustainability.

### Search Strategy

The databases searched were the Health Technology Assessment Database, MEDLINE, MEDLINE in Process, Embase, CINAHL, Conference Proceedings Citation Index, PsycINFO, and OpenGrey, from January 1, 1960, to December 31, 2023, a date range chosen to reflect the introduction of electronic or digitally supported health care. The search was conducted by the author IG in March 2024 and no language or location limits were applied. Medical Subject Headings (MeSH) terms and free-text words were used against the Sample, Phenomenon of Interest, Design, Evaluation, Research type framework [53] to collect relevant studies from the perspective of GPs in a primary care setting (see Multimedia Appendix 1). The search strategy is described in the PROSPERO registration and the previously published protocol [54]. The EndNote (Clarivate Plc) bibliographic software was used to organize all retrieved studies and remove any duplicates.

### Eligibility Criteria

Studies included were primary qualitative research conducted in primary care settings and published in English. The research had to have a focus on general or family practice and be qualitative in nature because we were specifically interested in GPs’ experience with and perspectives of CRs (see Multimedia Appendix 2 for detailed eligibility criteria).

### Study Selection and Data Extraction

We used the recommended full dual review approach, that is, 2 independent reviewers (IG and AP) screened the title, abstract, and then the full texts, as this has been shown to increase the number of eligible papers identified and more broadly minimize error in the selection process. The data were managed using an Excel spreadsheet (Microsoft Corporation), with any disagreements on the inclusion of a study consensually resolved through discussion with a third reviewer (IL). A data extraction form was designed to summarize study characteristics. The data extracted included information on study outcomes, study design, participants, and key findings.

### Quality Appraisal

The quality and bias of included papers were determined by the Critical Appraisal Skills Programme checklist for qualitative studies and the Mixed Methods Appraisal Tool for mixed method studies [55]. Although the Mixed Methods Appraisal Tool cautions against the approach of labeling studies at low, medium, and high risk of bias, we used a scoring system to determine this labeling, for ease of interpretation between the 2 different tools. The quality appraisal was undertaken independently by 2 authors (IL and IG) who between them agreed on the final value.

### Data Synthesis

The data were coded and managed via NVivo12 (Lumivero, LLC). We used a directed content analysis to populate the NASSS framework, based on the unconstrained matrix approach. This allowed the development and inclusion of constructs directly relevant to emergent themes within the established framework [56,57]. The analysis was conducted by IG and IL who both independently analyzed a sample of the same 3 papers. They then met to discuss their analysis and any discrepancies in the allocation of data before IL analyzed the remainder of the studies, with the final analysis and data allocation consensually agreed upon by all authors. In completing the analysis, we merged the domains of “Wider system” and “Embedding over time” due to the concurrence that arose in the data.

## Results

### Study Characteristics

**Figure 2:**
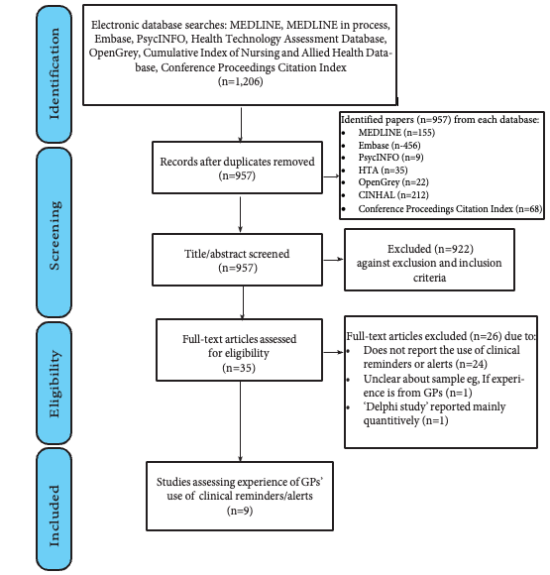
PRISMA (Preferred Reporting Items for Systematic Reviews and Meta-Analyses) flow diagram for the systematic review.

The screening and study selection are outlined in the PRISMA (Preferred Reporting Items for Systematic Reviews and Meta-Analyses) flow diagram (Figure 2; also see Multimedia Appendix 3). The study selection and data extraction templates can be found in Multimedia Appendix 4. Full-text studies were excluded because of duplication, or lack of detail on the care provider, setting, or digital tool (see Multimedia Appendix 5 for a detailed description). A total of 9 studies were included, 5 from the United Kingdom [58-62] and 1 each from Australia [63], Belgium [64], the Netherlands [65], and Norway [66]. Seven of these were considered high quality [58-62,65,66], 1 medium [64], and 1 low [63]. The papers were published between 2003 and 2021. The key characteristics of included studies and findings are summarized in Table 2. This includes the quality assessment (see Multimedia Appendix 6 for a detailed description). The directed content analysis enabled the mapping of the data within the NASSS framework. Within each domain, we determined a series of emergent constructs relevant to the implementation of CRs in primary care. These findings are summarized in Table 3 and presented below within each domain of the framework alongside exemplar quotes taken from the studies identified.

**Table 2:** Key characteristics and findings of identified studies.

Reference	Country of study	Study outcomes	Study design (quality of study)	Purpose of CR^a,b^	Participants/setting	Key findings
Ahearn and Kerr [63]	Australia	To determine GPs’^c^ perceptions of strengths and weaknesses of prescription systems	Focus groups (low)	Medication	14 GPs; 1 focus group in a rural area and 2 focus groups in an urban area	CRs affected prescribing behavior. GPs recognized the need for training. Considered helpful features of clinical decision support systems.
Bindels et al [65]	The Netherlands	To investigate the experiences of GPs with the “GRIF system” (automated test ordering combined with a feedback system)	Group discussion and interview (high)	Diagnosis	24 GPs; mean age 49 (SD 4.9) years, mean experience 19 (SD 7) years; males comprised 75%	The system increased attention to and promotion of guidelines.
Christensen and Grimsmo [66]	Norway	To study primary care physicians’ experiences of electronic prescription reminders	Focus groups; observations of doctor-patient encounters; and National Questionnaire Survey (high)	Medication	24 GPs (15 females + 9 males)	The ability to present relevant patient information and medical knowledge was appreciated.
Dikomitis et al [61]	United Kingdom	To obtain views from GPs who piloted the electronic risk assessment tools	Telephone interviews (high)	Diagnosis	23 GPs (13 males + 10 females)	Key issues over the integration of CRs into existing digital platforms.
Ford et al [59]	United Kingdom	To understand the features of individual clinical decision support systems that were barriers and facilitators to their use	Semistructured interview (high)	Medication	11 GPs (7 males + 4 females); age group 30-39 years, n=8; age group 40-49 years, n=2; and age group 50+ years, n=1	Issues emerged relating to the accuracy of CRs and their usability.
Jeffries et al [58]	United Kingdom	To understand the factors that influenced clinical decision support system implementation	Semistructured interviews (high)	Medication	14 GPs across 4 regions in the United Kingdom	There was a shared recognition of the need to use CRs and their potential to support patient care but felt CRs needed to be more relevant.
Heselmans et al [64]	Belgium	To assess users’ perceptions toward the EBMeDS system (a computer decision support system provided by EBPNet, a national computerized point-of-care information service in Belgium)	Survey (medium)	Management, medication, and diagnosis	Survey issued to 334 (Dutch-speaking) GPs	Reminders considered too general and inflexible, with questions over their reliability.
Holt et al [60]	United Kingdom	To investigate the implementation of the CRs in UK general practice	Semistructured interview (high)	Diagnosis	7 GPs (no characteristics described from the sample)	The advantages of CRs were recognized but with caveats over their specificity.
Slight et al [62]	United Kingdom	To examine the causes of prescribing and monitoring errors in general practice	Semistructured interviews and focus groups (high)	Medication	33 GPs participated in semistructured interviews and 46 contributed in focus groups	The need for prior training and improved information technology infrastructure and hardware.

^a^CR: clinical reminder.

^b^Medication, diagnosis, or ongoing management.

^c^GP: general practitioner.

**Table 3:** Summary of findings by NASSS^a^ domain.

NASSS domain and emergent constructs		Definition	Summary of findings (and review papers)
**Condition**			
	Medication	To support safe prescribing and medication adherence.	Issues accommodated by CRs^b^ included drug-drug interactions, drug allergies, and dose-related recommendations [58,59,62-64,66].
	Diagnosis	To support prompt and accurate diagnosis of a range of chronic and acute conditions.	Diagnostic use included both acute [60,61] and chronic conditions [64,65].
	Ongoing management	Providing reminders and alerts for the safe and successful long-term management of (chronically ill) patients.	CRs included references to reviews of patient medication, symptoms, and testing regimes [64].
**Technology**			
	Frequency and content of CRs	The number of CRs and the relevance and accuracy of their content.	The overabundance of CRs was described [59,61,63,64]. GPs^c^ described a preference for accompanying information that aided comprehension [59].
	Design and integration of CRs	The presentation and readability of CRs and their integration with existing clinical dashboards.	GPs described how CRs requiring a response disrupted their workflow [59,63]. A preference was expressed for CRs with clear color-coded designations of risk or otherwise readily understandable formatting [59]. Dropdown boxes were seen as overly complicated [62].
	Digital infrastructure	The capacity, capability, and compatibility of hardware and software systems and the interoperability of data.	The quality of connectivity and the capacity of practice software systems impacted the timely appearance of alerts, frustrating clinicians and increasing the risk of errors [59,60,62,66].
**Value proposition**			
	Overall quality and safety	CRs capable of presenting timely prompts with the necessary actions.	CRs were valued for their ability to provoke a decision [58,61,63], particularly in busy environments [59,60]. Their ability to act as educational tools was mooted [58]. The constant interruption of tasks by CRs was considered a risk to patient safety [59].
	Self-efficacy	CRs can provide reassurance for clinicians that they have not missed anything significant.	The increasing complexity of primary care meant that clinicians welcomed the additional support [59,61,63].
**Adopter**			
	Engagement, and compliance in CRs	The degree to which clinicians follow the recommendations of CRs and the factors that influence this decision.	There were concerns over the accuracy and relevance of CRs, with a need for more transparent evidence of their efficacy, as well as self-doubt if the CR was ignored [59,63].
	Digital literacy and acceptability	Ability to understand, use, and incorporate digital technologies in health care.	Recent cohorts of GPs were more familiar and trusting of digitalization [59]. There were concerns that training, experience, and context would be subjugated to computer-generated recommendations [59,61].
	Patient-clinician relationship	The impact on patient interactions, communication, and consultation behaviors.	CRs could disrupt consultations and inhibit patient communication [61,63].
**Organization**			
	Training in the purpose and use of CRs	Importance of providing adequate training and ensuring clinicians understand the purpose of CRs.	The importance of training was recognized when introducing CRs [58-61,63].
	Practice capacity and workflow	The capacity, workflow, and protocols of practice organizations.	Shortening consultation times precluded GPs from interacting with many of the CRs generated [58,60].
**Wider system adapting and embedding over time**			
	Adaptation over time	The ability of CRs to incorporate technological advances and reflect the evolving needs and preferences of patients and clinicians.	The need for greater and earlier engagement of software developers with GPs was described [58,63,65]. A number of suggestions for future CR design were shared across studies including better integration with existing software systems, patient-specific content, clear graphical formats, and the ability to customize frequency and content [59-61,63,67].
	Systemwide collaboration	Unified approaches to the long-term introduction of technology including procurement, infrastructure, and shared learning.	There was a recognized need for improved interoperability and shared data between settings and organizations [63,66] and for continued learning from peer experience was voiced [59].

^a^NASSS: Non-adoption, Abandonment, Scale-up, Spread, and Sustainability.

^b^CR: clinical reminder.

^c^GP: general practitioner.

### Condition

The studies identified described how CRs were used to optimize care and manage patient safety in 3 key areas: medication [58,59,62-64,66]; diagnosis, both broadly [64,65] and for specific conditions, namely, atrial fibrillation and stroke [60], and cancer [61]. CRs were also used in the ongoing management of chronically ill patients, providing reminders of reviews or tests [64].

### Technology

#### Challenges in Technological Aspects of CRs: Alerts, Design, and Infrastructure

Seven studies described issues relating to various technological aspects of CRs, specifically the frequency and content of alerts, their design and integration with existing interfaces, and the broader digital infrastructure and capability including the interoperability of data.

#### Frequency and Content of CRs

Four studies described how the inability to process a large number of alerts was a concern among GPs [59,61,63,64]. As one GP described:

There are so many things (...) so many things popping up, so many things prompting you. You don’t probably respond to all the prompts, because there’s a box here, a box there, a box everywhere, and you don’t see everything (...) It’s such a busy screen you don’t respond to everything...

61

The value of presenting accompanying patient-centered information alongside the clinical data of the CR was described in one study. As a GP elaborated:

It [the CR] actually comes up with a nice diagram that it can use with your patients, that’s got smiley faces of different colours to illustrate risk (...) it’s just a different way to help augment that communication with them. For things like that it’s quite helpful because patients are wary of tablets, quite rightly.

59

#### Design and Integration of CRs

Three studies described how pop-up CRs that required the checking of drop-down lists or otherwise had to be actioned before any next step were overly disruptive and actually inhibited their engagement with the CRs content [59,62,63]:

[when they appear]...you can’t go looking in the notes, you can’t input anything else, this is now taking priority, so you either suspend it and then re-open it and suspend it and re-open it.

59

#### Digital Infrastructure

The capacity of practice information technology (IT) systems and hardware was also a consideration, for example, some CRs slowed the operating speed of practice computers [59,60,62,66].

Our problem here is our computers are rubbish. They work really, really slowly (...) it takes a long time to come through so we scroll down an option, a pick list - it can pick the wrong thing! Which is very frustrating...

62

### Value Proposition

#### GPs’ Perspectives on CR Quality, Safety, and Educational Impact

GPs described their perspectives on the quality and safety of CRs, their potential to support continuing education and training, and their impact on self-efficacy.

#### Overall Safety and Quality

In 5 studies GPs reported that CRs were capable of improving patient safety by promoting reflection on their decision [58-61,63]:

...even if you immediately dismiss it, at least that millisecond you’ve thought about it, and I think that is going to be useful at some point, but for how many people I don’t know

61

GPs also appreciated that CRs could improve the quality of care by providing useful reminders of tasks or actions [59,60]:

...in a patient with diabetes where there are 17 things to do and I’ve just missed out two or three of them (...) it’s really nice to have “somebody” say “Oh, don’t forget, there’s the urine test, and you haven’t yet sent the patient for the eye test that you thought somebody else was doing, and it looks like nobody’s done after all so maybe get that done?”

59

GPs in one study reported how CRs could improve how they practice in the long term by their ability to promote and reinforce appropriate prescribing behaviors.

...and it’s actually educational, because a lot of them you know the warning before it comes up because you’ve seen it so many times so you’re already aware of it. So I mean some of the things I know now are because of it, so there is an educational element to it [...]

58

#### Self-Efficacy

There were conflicting accounts regarding the extent to which CRs enhance GPs’ confidence in practicing safely. In one study, GPs described how the presence of CRs was a source of reassurance, supporting their ability to provide safe care in the increasingly complex environment of modern primary care [61,63].

...work in general practice is getting bigger and bigger, and more work’s going to come to general practice. So, although it [the CR] can cause irritation, the ﬂip side of that is you can relax a little bit more and that you don’t have to remember absolutely everything (...)

63

In another study, a GP reflected upon how having to choose a predetermined response outside of their original decision led them to question their ability [59]:

...in order to dismiss it you have to select what action you’ve taken such as, well have you called an ambulance, have you sent them into hospital...you’re then panicking thinking gosh I don’t want, I don’t want to lie, no I haven’t sent them into hospital but is that a judgement, is that them saying because I haven’t done that I’m a rubbish doctor?

59

### Adopter

#### Factors Influencing GPs’ Engagement With CRs: Digital Literacy and Consultation Impact

From the perspective of those adopting CRs, GPs described the factors affecting their engagement with CRs, the influence of digital literacy, and the impact of CRs on their consultation.

#### Engagement and Compliance in CRs

In one study, GPs expressed hesitation in relying on a computer-generated algorithm [59]. Concerns surfaced in 3 studies over the accuracy and relevance of the recommendations associated with CRs, with examples including unrecognized contraindications [58,63,65]:

[drug-drug interactions] were mentioned in printed textbooks but were not picked up by the interaction checking facility of their prescribing software

63

One study described a preference for greater transparency between the clinical evidence and the presentation of the CR. As one GP described:

[We’re] inherently distrustful of anything that hasn’t got somebody saying “Oh! I’ve done this trial, and this trial, and we’ve used it!” and I think that’s what we’ve been taught at medical school - to be sceptical, until we’ve got the evidence.

59

#### Digital Literacy and Acceptability

The belief emerged that the new generation of GPs more familiar with digital technology in everyday life would be more accepting of computer-generated reminders [59].

I think to a certain extent there is a bit of a cultural change in the sense that the newer generation of doctors coming through are much more used to computers telling them things in their own lives and so they’re much more accepting of the idea that the computer might give them helpful information.

59

GPs in 3 studies described how the reliance on CRs risked undermining the accrual of contextual knowledge of a particular patient [59,61,67].

A lot of the patients who I kind of see regularly with chronic problems, I kind of know if they’re well or not because I know the patient and I just can tell if they’re themselves or if they’re not because you have a constant, you know, you lose that with a computer.

59

Related to this was the feeling that CRs were overly prescriptive and one GP felt that the value of their input was diminished relative to the diktats of the CR:

I think it feels frustrating...when the system is so process-driven that there’s no sort of autonomy of the GP to, you know, change it or, kind of, put in what’s relevant.

59

#### Patient-Clinician Relationship

Two studies reported that GPs understood the importance of focusing on patients during consultations, acknowledging that referral to, and interaction with, CRs can be an impediment to patient-centered interaction [61,63]:

I don’t use any separate clinical decision support tool in the consultation because of time problems, losing eye contact with patients

63

### Organization

#### Organizational Influences on CR Adoption: Training and System Capacity

In considering organizational influences, evidence emerged of the importance of training and the impact of existing practice capacity and systems.

#### Training and Purpose

GPs in 5 studies reflected on the importance of training not only to understand the purpose of CRs but also their functionality [58-61,63]. One GP bemoaned the typical lack of training before the introduction of digital tools:

...so, yeah things just happen [. . .] They often will appear before you have any training or knowledge of it whatsoever. Then if you’re lucky maybe there’s a launch event six months after you’ve already started using it.

58

#### Practice Capacity and Workflow

GPs noted how the pressure on their time restricted their ability to interact with CRs that take time to resolve [58,60]. As one GP explained:

We might have six or seven of them [reminders], and once you’ve got (...) worked through about three or four, and then there’s this anticoagulation...you think, well that’s another 5 or 10 minutes of consultation (...) so you leave it.

60

In another example, one GP likened the appearance of a CR to a colleague bursting through the door during a consultation:

It really is like an interruption, you know, no GP wants somebody to just burst in with the door opening or the phone ringing. In the same vein no GP really wants a big thing to just pop up on the screen that they didn’t call up.

59

### Wider System and Embedding Over Time

#### Enhancing CR Implementation: Systemwide Collaboration and Adaptation Strategies

The potential impact of improved systemwide collaboration was described, and a number of suggestions were made as to how CRs may be adapted to ensure more sustained implementation.

#### Systemwide Collaboration

It was suggested in 2 studies that more closely integrated data sets across settings would support the quality of the CRs [63,66].

I want a dynamic electronic health communication with the possibility of a written dialog and forwarding missing information.

66

The importance of sharing best practices and recommendations for certain CR systems between peers was also described:

I think there is a degree of sharing these kind of protocols and algorithms amongst GP practises and we certainly, you know, GPs talk to each other and say “oh yeah we’ve got this thing that alerts for that particular problem”, “oh, have you? Right great can we, can we bring it in?”

59

#### Adaptation Over Time

Three studies described how GPs would have liked greater engagement with the software developers in the design of the CRs [58,63,65]. Suggestions for ways in which CRs might be improved included the acquisition of patient-specific alerts, better integration into practice workflows, and customizability informed by GP feedback [59-61,63]. In terms of their design and content, there were also suggestions for the inclusion of additional details on dosage or color-grading CRs according to severity [63].

## Discussion

### General Findings

A total of 9 studies published since 2003 were identified, reporting research conducted in Europe, predominantly the United Kingdom, and Australia, 7 of which were judged to be of high quality. The findings from these studies were effectively categorized within the domains of the NASSS framework. Regarding Condition and Value Proposition, the use of CRs in diagnosis, prescribing, and the ongoing management of chronic conditions was seen as an effective means of maintaining or improving safe and high-quality care, albeit with contrasting effects on clinician self-efficacy. In terms of Technology attributes, the efficacy of CRs was found to be directly linked to their frequency, the presentation and accuracy of their content, and the speed and reliability of IT infrastructure and hardware. With respect to Adopter attitudes, GPs raised concerns about the accuracy of CRs and the potential loss of benefits derived from their clinical experience and consideration of individual patient contexts, in favor of algorithm-based decisions. From an Organization perspective, the importance of training and the ability of CRs to integrate seamlessly with existing workflows were emphasized. Finally, in the context of the Wider System, several suggestions were made to improve the usability of CRs, including the sharing of best practices and involving GPs earlier in the design process to ensure successful embedding over time.

### Specific Findings and Comparison With Existing Literature

#### Condition/Value Proposition

Managing the medications of a growing number of patients with multiple long-term conditions is a major contributor to GP workload in the United Kingdom and other countries [68,69]. The studies we identified suggest that CRs can play a positive role in supporting this management [58,59,62-64,66], and it is widely recognized that CRs present a valuable opportunity to reduce adverse drug reactions [70].

The potential of CRs to support diagnosis was highlighted in 4 of the identified studies [60,61,64,65]. This ability has been previously acknowledged as a significant advantage in primary care, where diagnostic errors are a critical concern, particularly for diseases with low incidence but high health risk [71-73].

Primary care is expected to take on an increasing role in managing chronic conditions across various countries and health systems, including the United Kingdom [74,75]. However, this capacity is often hindered by limited consultation times, high patient volumes, and inadequate communication with specialists [75,76]. In such contexts, CRs that draw on patient-specific data to remind GPs to test, review, or conduct periodic interventions appear highly valuable, though only 1 study explicitly examined this functionality [64].

The ability of CRs to prompt reflection on clinical decision-making was viewed positively in 5 of the studies [58-61,63]. The benefits of reflective practice on clinical decisions are well established, particularly when the feedback on decisions, as with CRs, is immediate [77].

#### Technology

The studies we identified confirmed that GPs felt overwhelmed by the number, complexity, and often poor functionality of CRs [59,61,63,64]. This cognitive overload caused by multiple appearances of CRs reflects previous research suggesting it can hinder clinician interaction with clinical decision support systems [18-20,78]. The cognitive burden associated with CRs also stems from the complexity of their content [59,62,63]. Therefore, CRs should be designed to accurately convey risk and relevant information in a clear, easily interpretable format that adheres to best practices in digital interface design [79-82]. Additionally, they should be better integrated into existing software and workflows [83,84].

Artificial intelligence has begun to be utilized in clinical decision support systems for individual conditions, and it is reasonable to assume it will play a role in refining the specificity of CRs in primary care [85]. However, efforts to minimize the negative impact of CRs, such as improving specificity [18] or allowing clinicians the autonomy to disable certain CRs [83], must remain mindful of patient safety implications [86].

The ability of GPs to respond promptly to CRs was also influenced by the speed and reliability of practice IT infrastructure and hardware [59,60,62,66]. Ensuring that existing IT systems can handle the growing prevalence and complexity of digital health care is widely acknowledged as a critical need in both high- and low- and middle-income countries. However, this challenge has yet to be universally addressed [87,88].

#### Adopter

GP concerns about CRs that enforce process-driven decisions at the expense of context and experience were described in 4 of the studies we identified [58,59,63,65]. Notably, lasting cynicism expressed by GPs who had experienced prior failures with CRs (described in 2 of the identified studies [59,63]) has been previously recognized in primary care, where digital interventions have failed to meet expectations [89].

A framework for technology developers has emerged that aims to enhance clinician confidence in clinical decision support systems by adhering to a predetermined set of criteria, focusing on understanding and sustaining trust among end users [90,91]. Evidence from one study we identified suggested that resistance to digital interventions may be lower among the generation of clinicians who grew up alongside the proliferation of digital technologies [59]. It is hoped that trust in CRs can be fostered through greater and earlier engagement with clinician end users by software developers and senior decision makers [92-94]. This aligns with long-standing recommendations for increased user involvement in the development of digital health tools [67,89].

The negative disruption of patient consultations by CRs was also highlighted [61,63]. It is already known that technology can interrupt the flow of conversation, reduce nonverbal communication cues, and compromise the overall quality of patient interactions [95,96]. Concerns expressed by GP participants in the identified studies reflect existing evidence that clinicians highly value uninterrupted in-person consultations, as they build patient trust and foster a constructive provider-patient relationship [97,98].

#### Organization

The lack of training emerged as a barrier to engagement and compliance with CRs in the studies we identified [58-61,63] and has previously been highlighted as central to their effective use, including sharing with GPs the processes behind their generation [99,100]. However, targeted training to enhance engagement with CRs has yet to be systematically implemented in the high-income countries where they are most prevalent [101].

The pressure on GPs to address increasingly complex care needs and rising patient expectations within shortening consultation times is widely acknowledged [102]. GPs in the identified studies reported that managing multiple CRs was unfeasible within the constraints of limited consultation times [58,60]. Some studies suggest that specific CRs, such as those supporting preventive care, could be delivered to patients before the consultation to alleviate the time pressure on GPs [103].

#### Wider System and Embedding Over Time

The need for systemwide coordination and collaboration in introducing CRs into primary care, as described in the review’s findings [59,63,66], has also been recognized in other health care settings [104-106]. Similarly, the calls for earlier clinician engagement in the design and development of CRs identified in this review [58,63,65] have been previously acknowledged [107-109], alongside the input of clinical informaticists [110]. While there are recognized challenges in engaging time-pressured GPs during the early stages of software development, sustainable adoption of health technology requires understanding its social and organizational contexts. This can only be achieved by involving end users throughout its design and development [111,112].

### Strengths and Limitations

The recognized systematic review methodology was applied to identify relevant papers [27,113], including the dual-review process [114]. The comprehensive NASSS framework successfully accommodated all of the data identified [26] and its use demonstrated both the transparency and rigor of our approach but also allowed us to structure and contextualize our data with existing research to avoid distorting our conclusions [28]. We acknowledge that there were a number of potential limitations. First, all the studies identified originated in high-income countries and were presented in English; however, we would suggest that the lessons learned have the potential for broader applicability for digital interventions in health systems of low- and middle-income countries that in reality share many of the same, if accentuated, barriers to technology-enabled care [111,112]. Second, it is possible that the use of an a priori framework might limit the analysis, with some data unable to be allocated within its confines; however, taking the unconstrained matrix approach suggested by Elo and Kyngäs [56] allowed the development and inclusion of emergent themes within the established framework. This meant we could maintain alignment with our established objectives and provide a systematic description of novel findings.

### Conclusions

If utilized correctly CRs can enhance patient safety, workflow management, preventative care, and reduce cognitive overload. Based on the findings of the review several recommendations can be made for the future design and implementation of CRs (see Table 4). These include improved graphical designs better suited to the purpose of the CR, more nuanced and patient-specific content, greater clinician autonomy regards if and when CRs are seen, and earlier and more consistent engagement with the workforce in their design and development.

**Table 4:** Recommendations for future CR^a^ design and implementation.

Domain	Recommendation
Technology	Visual-driven user interface to present information more effectively and reduce information overload. More directly applicable data (eg, dosage or risk). The capacity of the infrastructure.
Adopter	GP^b^-endorsed or GP-recommended CRs would promote and increase their uptake. Ensuring that CRs transparently reflect the latest clinical evidence.
Organization	Greater engagement with the workforce in the introduction of CRs. Additional training would help the uptake of CRs and using them effectively, accommodating the age and experience of the clinician. Enable organizations to better incorporate CRs in existing/novel workflows.
Wider systems	GP-stakeholders collaboration would enable feedback and co-design of alert utilization.
Adaptation	Alert customization would allow GPs to deactivate unnecessary options to change output and will provide flexibility.

^a^CR: clinical reminder.

^b^GP: general practitioner.

## Appendix

Multimedia Appendix 1 Search strategy.

Multimedia Appendix 2 Eligibility criteria.

Multimedia Appendix 3 PRISMA (Preferred Reporting Items for Systematic Reviews and Meta-Analyses) 2020 checklist (v17.12.24).

Multimedia Appendix 4 Study selection and data extraction templates.

Multimedia Appendix 5 Excluded studies.

Multimedia Appendix 6 Quality appraisal.

## Abbreviations

CR: clinical reminder
GP: general practitioner
IT: information technology
NASSS: Non-adoption, Abandonment, Scale-up, Spread, and Sustainability
PRISMA: Preferred Reporting Items for Systematic Reviews and Meta-Analyses

## Data Availability

This is a systematic review and the data it uses are in the public domain.
