# Supplementary material for: Understanding “Alert Fatigue” in Primary Care: Qualitative Systematic Review of General Practitioners Attitudes and Experiences of Clinical Alerts, Prompts, and Reminders
Source: J Med Internet Res. 2025 Feb 7;27:e62763. doi: 10.2196/62763 (PMC11845892; doi:10.2196/62763)
Supplement: Multimedia Appendix 1 [file jmir_v27i1e62763_app1.docx]

**Table S1a: Sample of the search term strategy terms using SPIDER**

| **S**ample | **P**henomenon of Interest | **D**esign | **E**valuation | **R**esearch type |
| --- | --- | --- | --- | --- |
| General practitioner* OR GP* | Alert fatigue* OR alarm fatigue* OR overridden alert* | questionnaire* OR survey* OR interview* OR focus group* | view* OR experience* OR opinion* OR attitude* OR perception* OR belief* OR feel* OR know* OR understand* OR challenges | Qualitative* OR Mixed method* |

**Table S1b: Ovid Search strategy for clinical alert fatigue experienced by GPs *(*Databased searched: MEDLINE, MEDLINE in Process, EMBASE, PsycINFO).**

| **No.** | **Search Term/Action** |
| --- | --- |
| 1 | exp Communication Barriers/ |
| 2 | barrier$.ti,ab. |
| 3 | enabler$.ti,ab. |
| 4 | facilitator$.ti,ab. |
| 5 | attitude$.ti,ab. |
| 6 | exp Attitude/ |
| 7 | exp attitude of health personnel/ |
| 8 | implementation.ti,ab. |
| 9 | physician acceptance.ti,ab. |
| 10 | perception$.ti,ab. |
| 11 | exp Perception/ |
| 12 | guideline$.ti,ab. |
| 13 | exp Guideline/ |
| 14 | exp Practice Guideline/ |
| 15 | exp Guideline Adherence/ |
| 16 | (lesson$ adj3 learned).ti,ab |
| 17 | negative impact.ti,ab. |
| 18 | quality improvement.ti,ab. |
| 19 | exp Health Services Research/ |
| 20 | exp Quality Improvement/ |
| 21 | negative response$.ti,ab. |
| 22 | useability.ti,ab. |
| 23 | user resistance.ti,ab. |
| 24 | challenge$.ti,ab. |
| 25 | difficult$.ti,ab. |
| 26 | failure$.ti,ab. |
| 27 | problem$.ti,ab. |
| 28 | OR/1-27 |
| 29 | alert$.ti,ab. |
| 30 | clinical reminder$.ti,ab. |
| 31 | exp Reminder Systems/ |
| 32 | clinical decision support system$.ti,ab. |
| 33 | exp Decision Support Systems, Clinical/ |
| 34 | computerized physician order entry.ti,ab. |
| 35 | exp Medical Order Entry Systems/ |
| 36 | electronic health record$.ti,ab. |
| 37 | exp Electronic Health Records/ |
| 38 | exp Medical Records Systems, Computerized/ |
| 39 | decision support system$.ti,ab . |
| 40 | exp Decision Making, Computer-Assisted/ |
| 41 | exp Diagnosis, Computer-Assisted/ |
| 42 | medical record system.ti,ab. |
| 43 | exp Information Systems/ |
| 44 | exp Medical Records/ |
| 45 | health informatic$.ti,ab. |
| 46 | exp Medical Informatics/ |
| 47 | reminder$.ti,ab. |
| 48 | exp Reminders/ |
| 49 | reminder system$.ti,ab. |
| 50 | diagnosis, computer assisted.ti,ab. |
| 51 | exp Clinical Alarms/ |
| 52 | warning$.ti,ab. |
| 53 | (alarm adj3 fatigue).ti,ab. |
| 54 | algorithm$.ti,ab. |
| 55 | exp Algorithms/ |
| 56 | tool.ti,ab. |
| 57 | (alert adj3 fatigue).ti,ab. |
| 58 | OR/29-57 |
| 59 | general practioner$.ti,ab. |
| 60 | 28 and 58 and 59 |
| 61 | limit 60 to yr="1960 - Current" |

(To be run in OVID with additional filter ‘Qualitative (maximises specificity))

**Table S1c: EBSCO, HTA, OpenGrey and Web of science search strategy for clinical alert fatigue experienced by GPs**

| Database | Search Strategy |
| --- | --- |
| EBSCO: Cumulative Index of Nursing and Allied Health (CINHAL) | " (Communication Barriers or barriers or enablers or facilitators or attitudes or attitude of health personnel or implementation or physician acceptance or perception or guideline or Practice Guideline or Guideline Adherence or negative impact or quality improvement or Health Services Research or Quality Improvement or negative responses or usability or user resistance or challenges or difficulties or failures or problems" ) AND ( general practitioner or general practice ) AND ( Alerts or clinical reminders or Reminder Systems or clinical decision support systems or Decision Support Systems or computerised physician order entry or Medical Order Entry Systems or electronic health record or Electronic Health Records or Medical Records Systems or decision support system or medical record system or Information Systems or Medical Records or health informatics or Medical Informatics or reminders or Reminders or reminder systems or computer assisted diagnosis or Clinical Alarms or warnings or alarm fatigue or algorithm or tool or alert fatigue)" |
| Health Technology Assessment (HTA) | (Alerts) OR (clinical reminders) OR (Reminder Systems) OR (clinical decision support systems) OR (Decision Support Systems) OR (computerised physician order entry) OR (Medical Order Entry Systems) OR (electronic health record) OR (Electronic Health Records) OR (Medical Records Systems) OR (decision support system) OR (medical record system) OR (Information Systems) OR (Medical Records) OR (health informatics) OR (Medical Informatics) OR (reminders) OR (Reminders) OR (reminder systems) OR (computer assisted diagnosis) OR (Clinical Alarms) OR (warnings) OR (alarm fatigue) OR (algorithm or tool) OR (alert fatigue)   AND  (Communication Barriers) OR (barriers) OR (enablers) OR (facilitators) OR (attitudes) OR (attitude of health personnel) OR (implementation) OR (physician acceptance) OR (perception) OR (guideline) OR (Practice Guideline) OR (Guideline Adherence) OR (negative impact) OR (quality improvement) OR (Health Services Research) OR (Quality Improvement) OR (negative responses) OR (usability) OR (user resistance) OR (challenges) OR (difficulties) OR (failures) OR (problems) |
| Opengrey | (qualitative) AND (Communication Barriers or barriers or enablers or facilitators or attitudes or attitude of health personnel or implementation or physician acceptance or perception or guideline or Practice Guideline or Guideline Adherence or negative impact or quality improvement or Health Services Research or Quality Improvement or negative responses or usability or user resistance or challenges or difficulties or failures or problems ) AND ( general practitioner or general practice ) AND ( Alerts or clinical reminders or Reminder Systems or clinical decision support systems or Decision Support Systems or computerised physician order entry or Medical Order Entry Systems or electronic health record or Electronic Health Records or Medical Records Systems or decision support system or medical record system or Information Systems or Medical Records or health informatics or Medical Informatics or reminders or Reminders or reminder systems or computer assisted diagnosis or Clinical Alarms or warnings or alarm fatigue or algorithm or tool or alert fatigue) |
| Web of science: Conference Proceedings Citation Index | **ALL=(Barriers) OR ALL=(enablers) OR ALL=(facilitator) OR ALL=(attitudes) OR ALL=(perception) OR ALL=(quality improvement) OR ALL=(user resistance) OR ALL=(challenges) OR ALL=(difficulty) OR ALL=(usability)**  AND  **(ALL=(general practitioner)) OR ALL=(general practice)**  AND  **(((((((((ALL=(alerts)) OR ALL=(clinical reminders)) OR ALL=(Reminder Systems)) OR ALL=(clinical decision support system)) OR ALL=(clinical decision support systems )) OR ALL=(computerized physician order entry)) OR ALL=(Medical Order Entry Systems)) OR ALL=(electronic health record)) OR ALL=(decision support system)) OR ALL=(Information Systems)**  **AND**  **ALL=(qualitative)** |
