# Supplementary material for: Understanding “Alert Fatigue” in Primary Care: Qualitative Systematic Review of General Practitioners Attitudes and Experiences of Clinical Alerts, Prompts, and Reminders
Source: J Med Internet Res. 2025 Feb 7;27:e62763. doi: 10.2196/62763 (PMC11845892; doi:10.2196/62763)
Supplement: Multimedia Appendix 2 [file jmir_v27i1e62763_app2.docx]

**Table S2: Eligibility criteria**

|  | **Inclusion criteria** | **Exclusion criteria** |
| --- | --- | --- |
| **Sample population** | •Health professionals including Doctors, Nurses and other health allied professionals  •Primary healthcare | •Professionals practising in secondary and tertiary healthcare  •Non-healthcare professionals |
| **Phenomena of Interest** | •The use of clinical reminder/alert. | •Any other phenomenon order than the use of clinical alert. Reminder will be excluded |
| **Design of study** | •All theoretical approaches  •Data collection method either focus groups or interviews (or both)  •Any analysis methodology | Quantitative designs |
| **Evaluation** | •Experiences of providers •Barriers and enablers of alert fatigue |  |
| **Research Type** | •Qualitative studies  •Mixed Methods | • Quantitative studies |
