# Supplementary material for: Understanding “Alert Fatigue” in Primary Care: Qualitative Systematic Review of General Practitioners Attitudes and Experiences of Clinical Alerts, Prompts, and Reminders
Source: J Med Internet Res. 2025 Feb 7;27:e62763. doi: 10.2196/62763 (PMC11845892; doi:10.2196/62763)
Supplement: Multimedia Appendix 4 [file jmir_v27i1e62763_app4.docx]

**Table S3a: Study Selection Form**

| Study information | Study ID |  |  |  |
| --- | --- | --- | --- | --- |
|  | Author |  |  |  |
|  | Year |  |  |  |
|  | Title |  |  |  |
| Inclusion Criteria  (Y/N) | Is the study either qualitative or mixed methods (containing qualitative elements)? |  |  |  |
|  | Is the study collecting a sample from GPs? |  |  |  |
|  | Are the GPs in primary care? |  |  |  |
|  | Is the study either qualitative or mixed methods (containing qualitative element)? |  |  |  |
|  | Does the study report experiences of the use of CRs from GPs? |  |  |  |
| Exclusion Criteria  (Y/N) | Is the study anything other than a qualitative study (for example quantitative)? |  |  |  |
|  | Are they a HCPsother than a GP? |  |  |  |
|  | Is the healthcare setting in secondary or tertiary care? |  |  |  |
|  | Does the study evaluate anything other than the experience of CR? |  |  |  |
|  | Include or exclude |  |  |  |
|  | Reason for exclusion |  |  |  |

**Table S3b: Data extraction form**

| **Study information** | Study ID |  |  |  |
| --- | --- | --- | --- | --- |
|  | Author |  |  |  |
|  | Year |  |  |  |
|  | Title |  |  |  |
| **Study characteristics** | Country of study |  |  |  |
|  | Setting |  |  |  |
|  | GP interview |  |  |  |
|  | Sample number in the study |  |  |  |
|  | Data collection method? |  |  |  |
|  | Types of CRs |  |  |  |
|  | Study analysis method |  |  |  |
|  | Healthcare professional perspectives only? |  |  |  |
| **Aims** | Study aims /purpose |  |  |  |
| **Study outputs** | Main themes |  |  |  |
|  | Recommendation/application |  |  |  |
