# Supplementary material for: Understanding “Alert Fatigue” in Primary Care: Qualitative Systematic Review of General Practitioners Attitudes and Experiences of Clinical Alerts, Prompts, and Reminders
Source: J Med Internet Res. 2025 Feb 7;27:e62763. doi: 10.2196/62763 (PMC11845892; doi:10.2196/62763)
Supplement: Multimedia Appendix 5 [file jmir_v27i1e62763_app5.docx]

**Table S4: Excluded studies at full-text screening**

| **Study title(ref)** | **Reason of exclusion** |
| --- | --- |
| Understanding GPs' views and experiences of using clinical prediction rules in the management of respiratory infections: a qualitative study(82) | Does not report the use of clinical reminders or alerts |
| Undertaking general practice quality improvement to improve cancer screening - a thematic analysis of provider experiences (83) | Does not report the use of clinical reminders or alerts |
| Barriers and facilitators to implementing a cancer risk assessment tool (QCancer) in primary care: a qualitative study (84) | Does not report the use of clinical reminders or alerts |
| Electronic clinical decision support tool for assessing stomach symptoms in primary care (ECASS): A feasibility study(85) | Does not report the use of clinical reminders or alerts |
| Future Health Today: exploring barriers and facilitators to implementation of a new technology platform for quality improvement in general practice.(86) | Does not report the use of clinical reminders or alerts |
| How do general practitioners access guidelines and utilise electronic medical records to make clinical decisions on antibiotic use? Results from an Australian qualitative study(87) | Does not report the use of clinical reminders or alerts |
| Using the electronic health record to build a culture of practice safety: Evaluating the implementation of trigger tools in one general practice (88) | Does not report the use of clinical reminders or alerts |
| Approaches to recording drug allergies in electronic health records: Qualitative study (89) | Does not report the use of clinical reminders or alerts |
| A newly implemented EMR: Effects on work flow and communication in family practice training.(90) | Does not report the use of clinical reminders or alerts |
| The development of a guideline implementability tool (GUIDE-IT): a qualitative study of family physician perspectives.(91) | Does not report the use of clinical reminders or alerts |
| The fungibility of time in claims of efficiency: The case of making transmission of prescriptions electronic in English general practice.(92) | Does not report the use of clinical reminders or alerts |
| Barriers to guideline implementation and educational needs of general practitioners regarding heart failure: a qualitative study(93). | Does not report the use of clinical reminders or alerts |
| What are the perceived learning needs of Australian general practice registrars for quality prescribing?.(94) | Does not report the use of clinical reminders or alerts |
| Barriers to the adoption of computerised decision support systems in general practice consultations: A qualitative study of GPs' perspectives.(95) | Does not report the use of clinical reminders or alerts |
| Clinical guidelines on depression: A qualitative study of GPs' views.(96) | Does not report the use of clinical reminders or alerts |
| DARTS 2000 online diabetes management system: Formative evaluation in clinical practice.(97) | Does not report the use of clinical reminders or alerts |
| Utilisation of computerised clinical guidance in general practice consultations.(98) | Does not report the use of clinical reminders or alerts |
| Evaluation of general practice computer templates: Lessons from a pilot randomised controlled trial.(99) | Does not report the use of clinical reminders or alerts |
| Information in general medical practice: a qualitative approach.(100) | Does not report the use of clinical reminders or alerts |
| User redesign, testing and evaluation of the Monitoring Risk and Improving System Safety (MoRISS) checklist for the general practice work environment.(101) | Does not report the use of clinical reminders or alerts |
| Exploring GPs' experiences of using diagnostic tools for cancer: a qualitative study in primary care.(102) | Does not report the use of clinical reminders or alerts |
| Computers in the examination room and the electronic health record: physicians' perceived impact on clinical encounters before and after full installation and implementation.(103) | Does not report the use of clinical reminders or alerts |
| Adoption of electronic medical records in family practice: the providers' perspective.(104) | Does not report the use of clinical reminders or alerts |
| Implementing an electronic medical record in a family medicine practice: communication, decision making, and conflict.(105) | Does not report the use of clinical reminders or alerts |
| Identifying and establishing consensus on the most important safety features of GP computer systems: e-Delphi study.(106) | Mainly reported outcome quantitatively rather than qualitatively. |
| Accessibility, usability, and usefulness of a Web-based clinical decision support tool to enhance provider–patient communication around Self-management TO Prevent (STOP) Stroke.(107) | Sample of study is unknown. For example, does not clarify whether experience is from GPs exclusively. |
