# Supplementary material for: Understanding “Alert Fatigue” in Primary Care: Qualitative Systematic Review of General Practitioners Attitudes and Experiences of Clinical Alerts, Prompts, and Reminders
Source: J Med Internet Res. 2025 Feb 7;27:e62763. doi: 10.2196/62763 (PMC11845892; doi:10.2196/62763)
Supplement: Multimedia Appendix 6 [file jmir_v27i1e62763_app6.docx]

**Table S5a: Detailed CASP quality appraisal**

| **Study information** | Study ID | 58 | 69 | 89 | 263 | 299 |
| --- | --- | --- | --- | --- | --- | --- |
|  | Author | Ford E.  Edelman N.  Somers L.  Shrewsbury D.  Lopez Levy M.  van Marwijk H.  Curcin V.  Porat T. | Jeffries M.  Salema N.-E.  Laing L.  Shamsuddin A.  Sheikh A.  Avery A.  Chuter A.  Waring J.  Keers R.N. | Ahearn M.D.  Kerr S.J. | Dikomitis L.  Green T.  Macleod U. | Slight S.P.  Howard R.  Ghaleb M.  Barber N.  Franklin B.D.  Avery A.J. |
|  | Year | 2021 | 2021 | 2003 | 2015 | 2013 |
|  | Title | Barriers and facilitators to the adoption of electronic clinical decision support systems: a qualitative interview study with UK general practitioners. | The implementation, use and sustainability of a clinical decision support system for medication optimisation in primary care: A qualitative evaluation. | General practitioners' perceptions of the pharmaceutical decision-support tools in their prescribing software. | Embedding electronic decision-support tools for suspected cancer in primary care: a qualitative study of GPs' experiences. | The causes of prescribing errors in English general practices: A qualitative study. |
|  |  |  |  |  |  |  |
| **CASP quality assessment.** | Was there a clear statement of the aims of the research? | The goal of the research is to support and optimise the design of future CDSSs by identifying factors influencing how or why GPs use these tools.   The importance of the research is to ensure CDSS are better designed and meets clinician needs. | The study aims to understand factors that influence primary care of a CDS system.  The importance of the research is to enhance quality and safety prescribing. | To explore how Australian GPs use PDS systems.  The importance of this research is to provide feedback to government, vendors and software developers. | To obtain views from GPs who use eRATs for suspected colorectal cancer.  The importance of this research is to find whether GPs were able to integrate these tools in their everyday practice, | To examine the causes of prescribing and monitoring errors in general practice.  The importance of this research is to provide recommendation for how they may be overcome. |
|  | Is the qualitative methodology appropriate? | Yes the research seeks to interpret GPs experience of the sue of CDSS, thus qualitative methodology is appropriate. | Yes the research seeks to interpret general practise staff experience of the use of CDS, thus qualitative methodology is appropriate. | Yes the research seeks to interpret GPs experience of the sue of PDS, thus qualitative methodology is appropriate | Yes the research seeks to interpret GPs experience of the use of eRATS thus qualitative methodology is appropriate. | Yes the research seeks to interpret general practise staff experience of what causes prescribing errors. |
|  | Was the research design appropriate to address the aims of the research? | The research justified the of thematic analysis as they wanted to approach with a realist ontology and an objectivist epistemology.  However, more details on why the method was chosen over other qualitative methods. | The research justified the use of Normalisation Process Theory (NPT) because it focuses upon the work people do to integrate intervention into practice. | Research design weren't justified and explained why they have used it. | The research justified the use of NPM because it is a robust conceptual model and it recognised for its flexibility in application. | The research used of reason's accident causational model; however, author does not justify the use of the thematic analysis. |
|  | Was the recruitment strategy appropriate to the aims of the research? | The researcher clearly explains the participants were recruited from BSMS and King's college.  Participants were appropriate to the study, as they were GPs who currently work in a general practise, thus can provide knowledge for this research   The researcher also states 1 GP refused to participate, however does not explain why and 1 recording failed | Researcher startes sampling frame was used and typology framework was developed based on demographic factors to recruit diverse groups of general practice staff.   Participants were appropriate to the study, as they were general practice staff, this can provide knowledge for this research.   The researcher clearly states why participants refused, due to time constraints and staffing commitments. | Recruitment was chosen by IT officers, who selected GPs with some knowledge of the system. However, this is inappropriate and can introduce bias    Participants were appropriate to the study, as they were GPs who currently work in a general practise, thus can provide knowledge for this research. | Participants in evaluation study was self-selected as the researcher stated it was not possible to do purposive sampling. Although, this may introduce bias, participants who were interviewed showed a diverse range in deprived areas to affluent areas.   Participants were appropriate to the study, as they were GPs who currently use the tool, thus can provide knowledge for this research. | Researcher used sampling strategy to recruit general practise, this allowed a diverse range of practices. However, the author acknowledges this and reports that the recruited practices had relatively high levels of interest.   Participants were appropriate to the study, as they were general practise staff who is involved in prescription. |
|  | Was the data collected in a way that addressed the research issue? | The data collection setting was justified as it was convenient for participants.   The data was collected via semi-structured interviews. The interviews were clearly outlined using topic guides.  However, it would have been beneficial if author explained why they chose this method  The method was not modified and data were audio recorded  No repeat interviews were performed. saturation of data was reached after 7th interviews. | Data collection setting was either in university premises or work place. This was due to convenience.   The data were collected via semi-structured interviews with topic guides.   Methods were not modified and interviews were audio recorded.  2 interviews taken place for each participant, with the 2nd interview approximately 12 months after 1st interview. | The data was collected via focus groups and asked a series of questions that the author has outlined.   No information of data saturation, or where the setting was.   Interviews were audio recorded. | Data collected via telephone interview with a topic guide and was guided by the research question  Interviews were audio recorded  No information on data saturation. . | Data collected through semi structures interviews and focus group with permission.   Setting of interview is unknown.   Interviews were audio recorded  Data saturation not mentioned. |
|  | Has the relationship between researcher and participants been adequately considered? | Data collection were conducted independently by a female freelance researcher, who had no relationship to participants.   The researcher used convenience sampling to recruit participants, however the researcher recognised this is a disadvantage as it resulted in more younger doctors participating | Researcher 'MJ' had no prior interaction with participants.   The author critically discusses biases with sampling as participants who agreed maybe more active in using systems. | Researcher does not provide any information about interaction with GPs. This causes bias as the interviewer and participants may have a relationship.  Also no sampling was used, IT officers selected GPs. In addition, author does not acknowledge this. | No information on the interaction or relationship with participants and interviewer.   The author critically discusses the limitation that participants were self-selected. However, the author states they did not exclusively end up with enthusiasts. | No information on the interaction or relationship with participants and interviewer.   Author considers implication of bias when recruiting, however conducted sampling strategy to cover range of locations. |
|  | Have ethical issues been taken into consideration? | Ethics approval were given by BSMS research governance and ethics committee   Consent was gained from each participant by verbal or physical consent (signing paper) | Ethical approval was granted by University of Manchester research ethic committee  Written Consent was gained from general practice staff. | Ethical issues have not been taken into consideration | Ethic approval by Hull York Medical School ethic committee | Ethical approval was obtained from Nottingham research ethics |
|  | Was the data analysis sufficiently rigorous? | Data analysis was done by using Braun and Clarke thematic analysis. The method was explained clearly as themes were refined by team and mapped closely to study aims.   In addition, themes and coding of transcript were undertaken by different team member. The researcher critically discussed the advantages and disadvantages  They also re-analysed transcript for further evidence | Data analysis was done by using NPT and clearly described the step undertaken.   Themes were mapped, integrated alongside the four NPT constructs   Several transcripts and themes were discussed with other researchers independent. | Data analysis was not explained properly, step by step  No thematic map or tree.   Themes were identified by preliminary Content analysis and further refined by an irritative process | Data analysis was done using NPM and clearly described each step.  Themes were mapped using the NPM.  Research teams held multiple regular meetings ro discuss themes from transcript. | Data analysis was done by using reason's accident causation model.  Themes were mapped and identified using this framework, and using constant comparison technique.   Negative cases were also examined to further refine explanation |
|  | Is there a clear statement of findings? | Findings suggest CDSS have not been designed for end user, practice and clinical context.  The researcher also discusses finding in a broader context, as they considered other factors that were not identified in their research | Findings suggest the use and sustainability of CDS was related to prescribers’ perception to relevance of alerts.  The researcher discusses findings compared to other studies, highlighting differences and similarities | Findings were not explicitly outlined; however the author identified several themes concerning with alerts and CDSS.   However, there was not much critical discussion happening for and against the authors findings, | Findings show that electronic decision tools were useful, however tool needs to be integrated in GP practices  The researcher discusses findings compared to other studies that reiterated their findings. | Findings found several categories of high-level error producing conditions  The researcher comparisons findings and study with the existing literature. |
|  | How valuable is the research? | The author states the relevance of their research in context of other studies in depth.   The researcher also considered other population such as US based GPs and makes recommendation to developers. | The author states the relevance of their research in context of other studies, as they explored the alert responses are related to different prescribers’ perception.   The researcher does not consider other population but acknowledges the different healthcare professionals. | The author states the relevance to stimulate discussion between software industry and relevant stakeholders.    The researcher does not consider other population but acknowledges the different population | Researcher states eRATs was the first time to incorporate cancer diagnosis with electronic system.   The researcher does not consider other population | The researcher made recommendations such as GP training, clinical governance and professional development.   Researcher does not consider other populations. |

**Table S5b: Detailed MMAT appraisal tool**

| **Study information** | Study ID | 90 | 167 | 531 | 586 |
| --- | --- | --- | --- | --- | --- |
|  | Author | Christensen T.  Grimsmo A. | Holt T.A.  Kirkpatrick S.  Hislop J.  Kearley K.  Mollison J.  Yu L.M.  Hobbs F.D.R.  Dalton A.R.H.  Marshall T.  Lasserson D.S.  Fitzmaurice D.  Fay M.  Qureshi N. | Heselmans A  Aertgeerts B  Donceel P  Geens S  Van de Velde S  Ramaekers D | Bindels R  Hasman A  Derickx M  Van Wersch JW  Winkens RA |
|  | Year | 2008 | 2018 | 2012 | 2003 |
|  | Title | Expectations for the next generation of electronic patient records in primary care: A triangulated study. | Barriers to a software reminder system for risk assessment of stroke in atrial fibrillation: A process evaluation of a cluster randomised trial in general practice. | Family physicians' perceptions and use of electronic clinical decision support during the first year of implementation. | User satisfaction with a real-time automated feedback system for general practitioners: a quantitative and qualitative study. |
| **Mixed Methods Quality Assessment** | 5.1)Is there an adequate rationale for using a mixed methods design to address the research question? | The author explains that a mixed method study is rational, as it would strengthen validity, credibility and repeatability of the study.. | The author references medical research council guidance on why mixed method should be used, however in the study it does not explain why, it just states that’s its recommended to evaluate | The authors explains that qual analysis is used to analyse factors in acceptance and use, where was quant analysis used to analysed computer-recorded interaction | Author does not explain why mixed method is rationale for this study, however it was appropriate of how they used questionnaire quantitively stats with qual analysis. |
|  | 5.2)Are the different components of the study effectively integrated to answer the research question? | Yes, the data collection is done by a triangulated design through interviews, questionnaires and focus groups, to create a complete 'image' | Yes, the data collection is done questionnaire responses and qualitative interview. Which were combined to give a better interpretation of findings. | Yes, the data collection is done questionnaire responses and qualitative interview. Which were combined to give a better interpretation of findings. | yes, data collection is done by questionnaire and qualitative interview, which were combined to give a better interpretation. |
|  | 5.3)Are the outputs of the integration of qualitative and quantitative components adequately interpreted? | Yes, the author integrates themes identified from interviews and quantitative results from questionnaires | Yes, the author integrates themes identified from interviews and quantitative results from questionnaires | Yes, the author integrates qual and quant results to identify factors. | Yes, the author integrates qual and quant results to identify GRIF acceptance and usability. |
|  | 5.4)Are divergences and inconsistencies between quantitative and qualitative results adequately addressed? | yes, there is no divergencies | yes, there is no divergencies | yes, there is no divergencies | yes, there is no divergencies |
|  | Do the different components of the study adhere to the quality criteria of each tradition of the methods involved? (Refer to MMAT appraisal tool criteria for the qualitative component 1.1 to 1.5) | | | | |
|  | 1.1)Is the qualitative approach appropriate to answer the research question? | Yes, as we are looking at experiences of GPs in the use of electronic patient records | Yes, as we are looking at experiences of GPs in the use of AURAS-AF | Yes, as we are looking at experiences of GPs in the use of CCDS | Yes, as we are looking at experiences of a GPs in the use of a GP tool |
|  | 1.2)Are the qualitative data collection methods adequate to address the research question? | Data collections methods included focus groups and interviews.  No information on how the qualitative data was recorded.   Furthermore, there was no clear justification of chosen data collection method. | Data collections methods included semi-structured interviews with topic guides.  Qualitative data was audio recorded.   Furthermore, there was no clear justification of chosen data collection method. | Data collections methods included questionnaire development.   Qualitative data was audio recorded.   Furthermore, there was no clear justification of chosen data collection method. | Data collections methods included interview. .   Qualitative data was audio taped.   Furthermore, there was clear justification of chosen data collection method. |
|  | 1.3) Are the findings adequately derived from the data? | The author uses quotes and gathered themes from group responses. However, qualitative analysis is unknown as they did not describe how they derived from data. | The author uses quotes and gathered themes from group responses and uses framework analysis to identify these themes from data. | The author does not use any quotes, but author states they have identified barrier and facilitators via the UTAUT model. | The author does not use any quotes, but author states the general consensus of GP experience |
|  | 1.4)Is the interpretation of results sufficiently substantiated by data? | Quotes provided to justify themes. | Quotes provided to justify themes. | no, no use of data collection linked | although no quotes have been used, links with data collection is identified |
|  | 1.5)Is there coherence between qualitative data sources, collection, analysis and interpretation? | yes there are clear links between interpretation and analysis. But coherence between data sources and collection was not as clear. | yes there are clear links between each component. | poor links between each component | yes there are clear links between each component. |
